# Self-Reported Waiting Times for Outpatient Health Care Services in Hungary: Results of a Cross-Sectional Survey on a National Representative Sample

**DOI:** 10.3390/ijerph18052213

**Published:** 2021-02-24

**Authors:** Óscar Brito Fernandes, Armin Lucevic, Márta Péntek, Dionne Kringos, Niek Klazinga, László Gulácsi, Zsombor Zrubka, Petra Baji

**Affiliations:** 1 Department of Health Economics, Corvinus University of Budapest, Fővám tér 8, H-1093 Budapest, Hungary; obritofernandes@uni-corvinus.hu; 2 Department of Public and Occupational Health, Amsterdam UMC, University of Amsterdam, Amsterdam Public Health research institute, Meibergdreef 9, 1105 AZ Amsterdam, The Netherlands; a.lucevic@amsterdamumc.nl (A.L.); d.s.kringos@amsterdamumc.nl (D.K.); n.s.klazinga@amsterdamumc.nl (N.K.); 3 Health Economics Research Center, University Research and Innovation Center, Óbuda University, Bécsi út 96/B, H-1034 Budapest, Hungary; pentek.marta@uni-obuda.hu (M.P.); gulacsi@uni-obuda.hu (L.G.); zrubka.zsombor@uni-obuda.hu (Z.Z.); 4 Corvinus Institute for Advanced Studies, Corvinus University of Budapest, Fővám tér 8, H-1093 Budapest, Hungary

**Keywords:** waiting time, patient experiences, outpatient care, EQ-5D-5L, Hungary

## Abstract

**(1) Background**: System-level data on waiting time in the outpatient setting in Hungary is scarce. The objective of the study was to explore self-reported waiting time for an appointment and at a doctor’s office. **(2) Methods**: An online, cross-sectional, self-administered survey was carried out in 2019 in Hungary among a representative sample (n = 1000) of the general adult population. Chi-squared test and logistic regression analysis were carried out to explore if socioeconomic characteristics, health status, or residence were associated with waiting times and the perception of waiting time as a problem. **(3) Results**: Proportions of 90%, 41%, and 64% of respondents were seen within a week by family doctor, public specialist, and private specialist, respectively. One-third of respondents waited more than a month to get an appointment with a public specialist. Respondents in better health status reported shorter waiting times; those respondents were less likely to perceive a problem with: (1) waiting time to get an appointment (OR = 0.400) and (2) waiting time at a doctor’s office (OR = 0.519). **(4) Conclusions**: Longest waiting times were reported for public specialist visits, but waiting times were favorable for family doctors and private specialists. Further investigation is needed to better understand potential inequities affecting people in worse health status.

## 1. Introduction

Across the Organization for Economic Co-operation and Development (OECD) countries, waiting times have been of interest for several years given their link with quality of care [1], including better care experiences and patient satisfaction [2]. In the last 15 years, many policies sought a meaningful decrease in waiting time across key services within the health care system, such as the introduction of maximum waiting time guarantees, the opportunity of being treated at another provider (including abroad) if waiting time limits are exceeded, and novel financing mechanisms [3,4].

Early international comparisons of waiting times across OECD countries were focused on elective hospital care, but in recent years this interest has broadened by considering ambulatory care [3]. An example of this broadening is the OECD’s proposed set of measures on patient experiences in ambulatory care (patient-reported experience measures, PREMs), where self-reported waiting time to get an appointment and at a doctor’s office before being seen by a doctor are highlighted [5]. These measures are part of the OECD’s framework for health systems’ performance assessment, and signal current concerns with value-based health care [6].

Hungary has a single payer, social insurance-based health care system with universal access [7,8,9]. Primary care is organized mainly through family doctors who are private entrepreneurs, and thus have decision power on how to best adapt their organization to its context and setting [8], including the ability to invest in information and communication technology infrastructure and software. The currently prevailing culture is one of service users and family doctors who are familiarized with an unscheduled approach to appointments, which is supported by the widely accepted standard practice of being seen by a family doctor on the same day. A referral system is in place for accessing secondary and tertiary care in the public sector; without a valid referral by a family doctor, the user may be asked to pay an out-of-pocket fee. Referral from a family doctor is compulsory for some specialties (e.g., cardiology, endocrinology, rheumatology), but not for others (e.g., otorhinolaryngology, gynecology, addictology). In recent years, the private sector has been an option to many; for example, in 2016, in Budapest, 60% of the residents accessed private health care services.

However, little is known about the responsiveness of the Hungarian health care system to its users’ expectations, needs, and experiences, including waiting times for health care services. Thus far, Hungary has not yet implemented a scheme to monitor waiting times across the health care system at large. Therefore, such information on waiting times is not available on a system level. Literature on waiting times in Hungary is scarce and focused on specific services and diseases [10,11,12,13,14]. Some of these studies found that waiting times for diagnosis and treatment in myeloma, colorectal cancer, and inflammatory bowel disease are comparable to those in international standards and/or in Western European countries. However, these studies presented the experiences of single treatment centers [10,11,12]. Often, data on self-reported unmet needs—available via the European Health Interview Survey (EHIS) and the European Union Statistics on Income and Living (EU-SILC)—are used to gauge proxy measures about waiting times, but results are not consistent.

To date, no study has reported on a system level about Hungarian waiting times for an outpatient consultation. However, such statistics are needed to strengthen person-centered care, to identify potential inequities, to support international comparisons, and set forth policies targeting increased access. Thus, this study aims to explore self-reported waiting time for an appointment and at a doctor’s office in the outpatient setting in Hungary, in addition to differences in waiting times by provider type, individuals’ sociodemographic characteristics, health status, and place of residence. We also examine the extent to which these self-reported waiting times were perceived as a problem by study participants, taking into consideration their sociodemographic characteristics.

## 2. Materials and Methods

### 2.1. Study Design

This was a cross-sectional descriptive study in Hungary, using data collected in 2019 via a web-based survey *‘Patient experiences in health care.’* The survey had three main modules: ‘eHealth literacy’ [15], ‘Shared decision-making’ [16], and ‘Patient-reported experience measures’ [17,18]. From the ‘Patient-reported experience measures’ module, we selected questions on waiting times as the focus, namely, on a set of patient-reported experience measures proposed by the OECD [5]. Data collection was performed by a survey company (Big Data Scientist Kft). Respondents were recruited from an online panel. The target sample size was 1000 respondents, defined based on rule of thumb. A disproportionate stratified random sampling was employed to reflect the characteristics of the general adult population of Hungary in terms of sex, age (by age group: 18–24, 25–34, 35–44, 45–54, 55–64, and 65 and over years), highest education level attained (primary, secondary, or tertiary), type of settlement (Budapest, other cities, or village), and region of residence (Nomenclature d’Unités Territoriales Statistiques (NUTS) level 1: Central Hungary, Great Plain and North, or Transdanubia). We used publicly available information of the Hungarian Central Statistical Office to characterize the distribution of the general adult population. Respondents were invited to participate in the survey via e-mail; thereafter, a web-link was provided granting access to the survey. Respondents were informed about the objectives of the survey and were required to provide consent before starting and at the end of the survey when submitting. Respondents’ answers were anonymized prior to analysis. Ethical approval was obtained by the Medical Research Council of Hungary (47654–2/2018/EKU).

### 2.2. Measures

#### 2.2.1. Waiting Time

The survey on patient experiences was based on a standardized set of questions set forth by the OECD, following earlier efforts of the Commonwealth Fund (e.g., International Health Policy Survey) and the Agency for Healthcare Research and Quality (e.g., the program Consumer Assessment of Healthcare Providers and Systems). The full set of questions endorsed by the OECD can be found in [5]. These questions focus on three areas: (1) access to care (unmet needs, waiting times to last outpatient visit); (2) patient experiences with the last outpatient visit (satisfaction with the length of the visit, communication with a doctor, and overall experience); and (3) respondents’ demographics. In this paper, we focus on the questions related to waiting times at the last outpatient visit during the 12 months preceding the survey and if waiting times were perceived as a problem by respondents. Other questions regarding patient experiences were analyzed in detail elsewhere [17,18].

The four questions analyzed in this study were the following: (i) ‘How quickly did you get an appointment to see this healthcare provider?’ (9 answer options, spanning from ‘same day’ to ‘91 days and longer’); (ii) ‘Was the time you waited for the appointment a problem for you?’ (1: yes); (iii) ‘On the actual day of the consultation, how long did you wait before you were actually seen?’ (7 answer options, from ‘up to 15 minutes’ to ‘more than 8 hours’); and (iv) ‘Was the time you waited to be seen a problem for you?’ (1: yes).

We presented and analyzed waiting times separately for the following three provider types: (1) general practitioner/family doctor at a doctor’s office; (2) specialist doctor at a public outpatient facility; and (3) specialist doctor at a private practice facility. The first two groups refer to health care services within a publicly financed health care system, and the third group refers to privately purchased services in the private sector.

#### 2.2.2. Sociodemographic Characteristics and Health Status

We collected information on sociodemographic characteristics: sex, age group, highest educational level attained (primary, secondary, tertiary), marital status (1: married/in a relationship), employment status (1: having a paid job), household income quintile (1: lowest income quintile), type of settlement (capital, other cities, village), NUTS 1 region of residence, and health status. Health status was measured with the EQ-5D-5L instrument, which is a generic health status measure that comprises five dimensions: mobility, self-care, usual activities, pain/discomfort, and anxiety/depression [19]. Respondents were asked to indicate their current health state that day in each of those dimensions using a 5-level scale (1: no; 2: slight; 3: moderate; 4: severe; and 5: unable/extreme problems). We used the tariffs for England in our study (value range: −0.285 to 1) to calculate EQ-5D-5L index score, given that tariffs for Hungary were not available at the time of the study. In the second part of the EQ-5D-5L instrument, respondents were asked to value their current health that day on an EQ VAS, a vertical a 0–100 visual analogue scale (0—worst, 100—best health state the respondent could imagine).

### 2.3. Statistical Analysis

First, we presented the distribution of self-reported waiting time to get an appointment and to be seen by a doctor, across type of providers. For each type of provider, we used the chi-squared test to assess the association between sociodemographic variables, health status, residence, and self-reported waiting times. Second, we used the chi-squared test to explore the association of respondents’ socioeconomic characteristics, health status, provider type, and appointment and office waiting times with the following outcome variables: (1) waiting more than 30 days for a public specialist appointment (family doctor and private specialist visits were excluded from the analysis due to the differences in organization and access to these services; the share of those who waited more than 30 days for an appointment was low, i.e., 5% and 4%, respectively (See Table 2)); (2) waiting more than 2 h at the doctor’s office; (3) reporting waiting time for an appointment as a problem; and (4) reporting waiting time at the doctor’s office as a problem. Thereafter, we used logistic regression to explore potential determinants of those outcome variables. The following covariates were included in the analyses and logistic regression models: waiting time to get an appointment, waiting time at a doctor’s office, provider type, sex, age groups, education, marital status, employment status, household income quintile, type of settlement, region of residence, and health status. We used the EQ VAS (73.9; SD = 19.6) and the EQ-5D-5L (0.858; SD = 0.163) mean values as cutoffs to classify respondents into two groups (below/above mean). Crude odds-ratios are presented in Online Supplement Tables S1 and S2.

All analyses were conducted in Stata 16. A *p*-value < 0.05 was considered statistically significant.

## 3. Results

### 3.1. Respondents’ Characteristics

The online sample of 1000 respondents represented well the Hungarian adult population considering sex, age, type of settlement, and region of residence. A comparison of the distribution of the sample with the Hungarian general population based on the last census in 2011 is available in [15]. Of the 1000 participants, 695 individuals had an outpatient consultation at a family doctor, public specialist, or private specialist within the 12 months preceding data collection. Of these 695 participants, 664 answered all four questions on waiting time. This subsample was considered for further analyses (Table 1). Forty-five percent of the respondents (*n* = 298) had their last consultation with a family doctor, 44% (*n* = 290) with a specialist doctor in the public sector, and 11% (*n* = 76) with a specialist doctor in the private sector. Most respondents were women (55%), between 35 and 64 years old (47%), without a paid job (51%), and living in urban areas (79%).

### 3.2. Waiting Time to Get an Appointment

Waiting time to get an appointment followed different patterns by type of provider (Table 2). In the subgroup of respondents who visited a family doctor (*n* = 298), the majority (*n* = 204, 68%) reported that they were seen on the same day, and 90% (*n* = 268) were seen within 1 week. Of those respondents who visited a public specialist (*n* = 290), 16% (*n* = 46) were seen on the same day, 41% (*n* = 119) within a week, 50% (*n* = 144) within two weeks, and 67% (*n* = 193) within a month. Considering the respondents who had a private specialist appointment (*n* = 76), 7% (*n* = 5) were seen on the same day, 64% (*n* = 49) were seen within a week, 79% (*n* = 60) within two weeks, and 88% (*n* = 67) within a month. Most frequent self-reported waiting time to get an appointment with a family doctor was ‘same day’ (68%), ‘same day’ and ‘15–30 days (more than two weeks)’ for a public specialist appointment (16% and 17% respectively), and ‘2–5 days (couple of days)’ for a private specialist appointment (36%).

Table 3 presents the share of respondents who waited longer than 30 days for a public specialist appointment. Results suggested significant associations with employment status (chi^2^ = 7.6, *p* = 0.006) and health status (chi^2^ = 6.0, *p* = 0.014). Regarding the former, respondents without a paid job reported waiting longer than 30 days for an appointment, relative to those with a paid job (39.5% vs. 23.9%); similarly, respondents with worse health status (EQ VAS below average) reported waiting longer than 30 days for an appointment relative to those with better health status (40.4% vs. 26.8%). Association with income quintile was also significant (chi^2^ = 12.2, *p* = 0.016), however, no specific tendency was observed.

Multivariate logistic regression results (Table 3) show that respondents from age group 35–44 (odds-ratio (OR) = 0.312, 95% confidence interval (CI): 0.098–0.993), from the 4th quintile (OR = 0.278, 95% CI: 0.096–0.810), and from the Great Plain and North (OR = 0.347, 95% CI: 0.137–0.879) were significantly less likely to report waiting longer than 30 days for an appointment with a public specialist.

### 3.3. Waiting Time at a Doctor’s Office

Distributions of self-reported waiting times at a doctor’s office were very similar for family doctor and public specialist appointments (Table 4). For example, the share of respondents that self-reported to have waited at a doctor’s office up to 15 min was 21% for a family doctor and 19% for a public specialist. In addition, waiting time was up to 60 min for 69% of the family doctor visits, and 62% of the public specialist consultations. However, the distribution of self-reported waiting time at a private specialist office was more right-skewed, compared with other type of providers. Of those respondents who had a private specialist appointment, 59% (*n* = 45) self-reported to have waited up to 15 min and 91% (*n* = 69) were seen within 60 min.

The results showed a significant association with provider type (chi^2^ = 12.4, *p* = 0.002), where 17.6% (*n* = 51) of the respondents reported to have waited longer than 2 h at a public specialist’s office, contrasting with 12.1% for a family doctor (*n* = 36), or 2.6% (*n* = 2) for a private specialist (Table 5). We also found a significant association with health status (chi^2^ = 3.9, *p* = 0.049) with a higher share of respondents in worse health status reporting waiting longer than 2 h at a doctor’s office (16.6% vs. 11.3% for respondents below and above average EQ VAS, respectively).

Respondents who visited a private specialist (OR = 0.101, 95% CI: 0.021–0.479) were less likely to wait more than two hours at the doctor’s office, relative to those who visited a public specialist (Table 5).

### 3.4. Waiting Time Perceived as a Problem

Among the respondents who were not seen on the same day by a doctor (*n* = 409), 103 (25.2%) reported that the waiting time to get an appointment was a problem for them (Table 6). Perceiving waiting time to get an appointment as a problem was significantly associated with the waiting time to get an appointment (chi^2^ = 51.5, *p* < 0.001), with waiting time at the doctor’s office (chi^2^ = 47.1, *p* < 0.001), sex (chi^2^ = 7.4, *p* = 0.007), and health status (EQ-5D-5L: chi^2^ = 6.5, *p* = 0.011; and EQ VAS: chi^2^ = 7.8, *p* = 0.005), where a greater share of women and respondents in worse health status perceived waiting time to get an appointment as a problem.

Respondents who waited more than two weeks had significantly higher odds of reporting the waiting time to get an appointment as a problem, relative to respondents who had an appointment the following day (next day appointment) (Table 6). Those respondents whose EQ-5D-5L score was above mean (indicating better health status at the time of the survey), were less likely to perceive the waiting time for an appointment as a problem (OR = 0.400, 95% CI: 0.210–0.763). Men were also less likely to report that waiting time was a problem for them than women (OR = 0.321, 95% CI: 0.156–0.662). Furthermore, respondents with a paid job had significantly greater odds of reporting waiting time to get an appointment as a problem relative to those respondents without a paid job (OR = 2.237, 95% CI: 1.018–4.917).

Among the respondents who waited longer than 15 min at the doctor’s office (n = 501), 178 (35.5%) reported that waiting time at the office was a problem for them (Table 7). Results showed significant associations between perceiving waiting at a doctor’s office as a problem and the waiting time at the office (chi^2^ = 83.3, *p* < 0.001), age (chi^2^ = 20.5, *p* = 0.001), income (chi^2^ = 11.8, *p* = 0.019), and health status (chi^2^ = 4.3, 0.038).

Multivariate logistic regression showed that in contrast to those who waited between 15 and 30 min, respondents that waited longer had significantly higher odds of perceiving the time they waited as a problem (Table 7). Compared to those over the age of 65, respondents between the ages of 25 and 34 were significantly more likely to report that waiting time at the doctor’s office was a problem for them (OR = 5.329, 95% CI: 2.090–13.59), whereas respondents in better health status were less likely to perceive waiting at a doctor’s office as a problem (OR = 0.519, 95% CI: 0.307–0.879).

## 4. Discussion

We investigated two types of waiting time in outpatient care in Hungary: waiting time to get an appointment and at a doctor’s office, at three types of providers: family doctor, and outpatient specialists in the public and private sector. We also explored when waiting times were reported by respondents as a problem. Our results indicated differences in self-reported waiting time distributions, between and within type of provider. Respondents with worse health status were more likely to wait longer and report that waiting time was a problem. Respondents with a paid job were more likely to perceive waiting time as a problem, relative to those without a paid job.

### 4.1. Waiting Times in the Outpatient Setting

Most respondents (68%) who had a family doctor appointment were seen on the same day and 90% within a week. In Hungary, citizens may go to their family doctor’s office with no previous appointment, which partly explains the large share of respondents who have reported a same-day appointment. We assumed that the remaining respondents got a scheduled appointment, in particular those who go to a family doctor regularly (e.g., people with chronic conditions, prevention check-up). These waiting times to get an appointment with a family doctor were relatively short, particularly when considering the generalized shortage of family doctors in Hungary, especially in rural areas. 

The waiting time to get a public specialist appointment was concentrated between 2 and 30 days, with 33% of respondents reporting to have waited longer than 30 days. Based on the same set of OECD questions and a comparable methodology, similar or somewhat lower shares were reported from France (36%), the Netherlands (25%), Germany (25%), and Switzerland (23%); and much higher proportions from the UK (41%), Norway (61%), and Sweden (52%). The results found in this study may be partly explained by the referral system in place in Hungary, in which family doctors act as gatekeepers to specialized care. In addition, relatively favorable waiting times in the public sector (family doctor and public specialist) could partly be explained by the number of doctor consultations per capita, in all settings (Hungary: 10.7 in 2018; OECD average, 6.6), whereas the number of physicians and nurses per capita are below the OECD averages (physicians: 3.4 vs. 3.5; nurses: 6.6 vs. 8.8). A below-average number of personnel providing above average number of consultations might negatively affect the quality of care and the responsiveness of providers to people’s care needs, expectations, and experiences. Nevertheless, based on the results of our previous studies based on the same questionnaire and sample, respondents were overall satisfied with the quality of their last outpatient visit; only 11.5% of respondents reported that the doctor did not spend enough time in consultation, and 12.6% reported that the doctor did not give an opportunity to ask questions or raise concerns. Yet, longer waiting times in the public system could still potentially be reduced by a greater integration of care across actors of the care system, where digitalization could facilitate an effective use of resources and reduce any unnecessary navigations of users across the care system [20,21,22,23].

In contrast, the distribution of waiting time to get an appointment at a private specialist was right-skewed. This partly signals that those who attended private specialists (and paid out-of-pocket for care at the private sector) wanted to be examined sooner than it would have been possible in the public system. We also hypothesize that in the profit-oriented private sector, users’ expectations, needs, experiences, and satisfaction are greatly taken into consideration, even if measuring such performance indicators is not fully anchored in a comprehensive strategy of measuring the performance of the health system [24].

The distributions of waiting time at a doctor’s office were similar at a family doctor’s and public specialist’s office, whilst at a private specialist’s office was right-skewed, where 80% of those who attended such an appointment waited less than 30 min at the office. Given that there is not a culture of systematically collecting patient-reported outcomes and experience measures, the extent to which shorter waiting times may be signaling inadequate time spent with a patient in consultation in not known. It could be the case that consultations in the private sector are too profit-oriented with negative consequences for care quality.

### 4.2. Waiting Times Reported as a Problem

People with worse health status reported longer waiting times and were also more likely to perceive waiting time as a problem. This is an alarming finding given that longer waiting times can further deteriorate one’s health. Nevertheless, it is also possible that acute visits are prioritized over scheduled routine follow-up appointments of chronic patients. Respondents who had a paid job were also twice as likely to perceive waiting time to get an appointment as a problem, relative to those without a paid job. A possible explanation for this finding is that respondents with a paid job value their good health more relative to those without a job, linking investments in health with greater productivity and earnings. These findings are aligned with those reported in other studies, where socioeconomic status was associated with how people experienced waiting time across the health care system [25,26,27].

Our results did not suggest many significant associations between respondents’ sociodemographic characteristics and waiting time measures, including perceiving waiting time as a problem. This could signal that perception of waiting time as a problem was controlled for actual reported waiting times (i.e., those who wait longer more often perceive waiting time as a problem). Furthermore, considering the cultural context, the weight citizens assign to waiting time attributes is relatively small compared to that of other features of health care services (e.g., a doctor providing easy to understand explanations, or involving the patient in decision-making about care/treatment). Recent evidence from a discrete choice experiment in which the full sample of the survey *‘Patient experiences in health care’* was used, showed that respondents’ willingness to pay varied from €4.38 to wait an hour less at a doctor’s office to €5.46 for one week reduction in the waiting time for an appointment; by comparison, other attributes of the care experience were highly valued (e.g., on average, respondents were willing to pay €36.13 more to have an appointment with a doctor that spends enough time in consultation with a patient) [28].

### 4.3. Study Limitations

Our study was based on data collected via a self-administrated online survey, which means that people without internet access could not participate (selection bias). Nevertheless, our survey sample well represented the Hungarian adult population considering sex, age, type of settlement, and region of residence. However, people with primary education or less were underrepresented in the survey. The proportion of respondents who declined to answer waiting time questions (participation bias) or reported that they did not remember were below 2%, which should not affect our results. We should also stress that some recall bias may have occurred in answering waiting time questions, given that we asked respondents about their experience on the last visit in the previous 12 months. Furthermore, we considered only those respondents who had a visit, and did not account for forgone visits due to a long waiting time for the appointment or at the office. Finally, we did not collect more information on the content and urgency of the visit (routine appointment/urgent visit, acute/chronic health problem, with/without a referral), which could largely influence waiting times.

## 5. Conclusions

To our knowledge, this is the first study reporting on waiting times for outpatient services in Hungary on a system level using a set of standardized questions. Our findings suggest that waiting times were favorable for family doctors and private specialists, however longer waiting times were reported for public specialist visits, where about one-third of the respondents waited longer than one month for an appointment. Respondents in worse health status reported longer waiting times to get an appointment and were more likely to perceive waiting time as a problem. This finding signals that further investigation of this topic is needed to better understand the extent and magnitude of waiting times on citizens’ health and well-being, with special focus on the most vulnerable.

Our work may also contribute to the strengthening of a health system performance assessment culture in the Hungarian health care system, anchored on the use of international standardized measures. In this study, we used a set of patient-reported experience measures endorsed by the OECD, which could provide reliable information for governance mechanisms to pinpoint where improvements are needed the most. In addition, the use of such standardized measures could enable further international comparison and could be used for benchmarking purposes. 

## Figures and Tables

**Table 1:** Sociodemographic characteristics of the sample by type of provider.

	Family Doctor		Public Specialist		Private Specialist		Total	
	*n*	%	*n*	%	*n*	%	*n*	%
**Sex**								
Men	146	49	125	43	28	37	299	45
Women	152	51	165	57	48	63	365	55
**Age group (years)**								
18–24	38	13	22	8.0	5	7.0	65	10
25–34	51	17	31	11	33	43	115	17
35–44	50	17	48	17	21	28	119	18
45–54	43	14	36	12	4	5.0	83	13
55–64	55	18	49	17	3	4.0	107	16
65+	61	21	104	36	10	13	175	26
**Employment status**								
Without a paid job	132	44	177	61	27	36	336	51
With a paid job	166	56	113	39	49	64	328	49
**Education**								
Primary	102	34	92	32	15	20	209	32
Secondary	107	36	103	35	25	33	235	35
Tertiary	89	30	95	33	36	47	220	33
**Region of residence**								
Central Hungary	96	32	105	36	32	42	233	35
Great Plain and North	108	36	108	37	20	26	236	36
Transdanubia	94	32	77	27	24	32	195	29
**Type of residence**								
Village	62	21	67	23	14	18	143	21
Capital	63	21	64	22	17	22	144	22
Other cities	173	58	159	55	45	59	377	57
**Income quintile**								
1 (lowest)	60	24	55	22	11	17	126	23
2	38	15	42	17	6	10	86	15
3	51	21	50	20	10	16	111	20
4	63	25	46	19	22	34	131	23
5 (highest)	36	15	54	22	15	23	105	19
**EQ-5D-5L index** (mean: 0.858)								
Below mean	88	30	114	39	23	30	225	34
Above mean	210	70	176	61	53	70	439	66
**EQ VAS** (mean: 73.9)								
Below mean	100	34	141	49	24	32	265	40
Above mean	198	66	149	51	52	68	399	60

**Table 2:** Waiting time to get an appointment by type of provider.

	Family Doctor (*n* = 298)			Public Specialist (*n* = 290)			Private Specialist (*n* = 76)		
	*n*	%	Cumulative	*n*	%	Cumulative	*n*	%	Cumulative
**Waiting Time to Get an Appointment**									
0 days (same day) *	204	68%	68%	46	16%	16%	5	7%	7%
1 day (next day)	29	10%	78%	11	4%	20%	7	9%	16%
2–5 days (couple of days)	29	10%	88%	32	11%	31%	27	36%	51%
6–7 days (just less than a week)	6	2%	90%	30	10%	41%	10	13%	64%
8–14 days (more than a week)	6	2%	92%	25	9%	50%	11	14%	79%
15–30 days (more than two weeks)	10	3%	95%	49	17%	67%	7	9%	88%
31–60 days (more than one month)	5	2%	97%	41	14%	81%	6	8%	96%
61–90 days (more than two months)	5	2%	99%	32	11%	92%	2	3%	99%
91 days or longer (more than three month	4	1%	100%	24	8%	100%	1	1%	100%

* Same day includes those who did not make an appointment and went directly to a family doctor (n = 94), a public specialist (*n* = 14), or a private specialist (*n* = 1).

**Table 3:** Share of respondents who waited more than 30 days for an appointment with a public specialist and multivariate logistic regression results.

	Share of Respondents who Waited More than 30 Days for an Appointment with a Public Specialist %	Multivariate Logistic Regression: Waited More than 30 Days for an Appointment with a Public Specialist OR (95% CI)
**Total**	33.4%	-
**Sex**	Chi^2^ = 0.2 (*p* = 0.649)	
Female	34.5%	(Baseline)
Male	32.0%	0.613 (0.320–1.177)
**Age group**	Chi^2^ = 10.3 (*p* = 0.067)	
18–24	27.3%	0.522 (0.145–1.879)
25–34	22.6%	0.406 (0.106–1.563)
35–44	18.8%	0.312 * (0.0980–0.993)
45–54	38.9%	1.056 (0.338–3.297)
55–64	36.7%	0.532 (0.202–1.403)
65+	41.3%	(Baseline)
**Marital status**	Chi^2^ = 0.1 (*p* = 0.820)	
Not married	34.3%	(Baseline)
Married	33.0%	1.048 (0.573–1.916)
**Employment status**	Chi^2^ = 7.6 (*p* = 0.006) **	
Without a paid job	39.5%	(Baseline)
With a paid job	23.9%	0.516 (0.228–1.170)
**Education**	Chi^2^ = 1.7 (*p* = 0.419)	
Primary	38.0%	(Baseline)
Secondary	29.1%	0.635 (0.310–1.302)
Tertiary	33.7%	0.743 (0.308–1.792)
**Income quintile**	Chi^2^ = 12.2 (*p* = 0.016) *	
1 (lowest)	41.8%	(Baseline)
2	26.2%	0.409 (0.142–1.171)
3	46.0%	1.251 (0.513–3.049)
4	17.4%	0.278 * (0.0957–0.810)
5 (highest)	40.7%	0.804 (0.291–2.221)
**EQ-5D-5L**	Chi^2^ = 0.2 (*p* = 0.634)	
Below median	35.1%	(Baseline)
Above median	32.4%	1.211 (0.662–2.212)
**EQ VAS**	Chi^2^ = 6.0 (*p* = 0.014) *	
Below median	40.4%	-
Above median	26.8%	-
**Settlement**	Chi^2^ = 2.0 (*p* = 0.362)	
Village	26.6%	(Baseline)
Capital	36.5%	0.468 (0.155–1.413)
Other cities	32.8%	1.024 (0.479–2.191)
**Region**	Chi^2^ = 1.2 (*p* = 0.562)	
Central Hungary	35.2%	(Baseline)
Great Plain and North	29.6%	0.347 * (0.137–0.879)
Transdanubia	36.4%	0.704 (0.282–1.758)
**Observations**	290	247
**Wald Chi^2^ (*p*)**	-	36.57 (*p* = 0.0090)
**Pseudo R^2^**	-	0.1193

OR: odds ratio. Robust 95% confidence intervals (CI) in parentheses. * *p* < 0.05, ** *p* < 0.01, *** *p* < 0.001.

**Table 4:** Waiting time at a doctor’s office by type of provider.

	Family Doctor (*n* = 298)			Public Specialist (*n* = 290)			Private Specialist (*n* = 76)		
	*n*	%	Cumulative	*n*	%	Cumulative	*n*	%	Cumulative
**Waiting Time at a Doctor’s Office**									
Up to 15 min	63	21%	21%	55	19%	19%	45	59%	59%
More than 15 and up to 30 min	75	25%	46%	61	21%	40%	16	21%	80%
More than 30 and up to 60 min	69	23%	69%	63	22%	62%	8	11%	91%
More than 1 and up to 2 h	55	18%	88%	60	21%	82%	5	7%	97%
More than 2 and up to 3 h	32	11%	99%	42	14%	97%	1	1%	99%
More than 4 and up to 8 h	4	1%	100%	9	3%	100%	1	1%	100%
More than 8 h	0	0%	100%	0	0%	100%	0	0%	100%

**Table 5:** Share of respondents who waited more than 2 h in the doctor’s office and multivariate logistic regression results.

	Share of Respondents Waited More than 2 h in the Doctor’s Office	Multivariate Logistic Regression: Waited More than 2 h at the Doctor’s Office OR (95% CI)
**Total**	13.4%	-
**Provider**	Chi^2^ = 12.4 (*p* = 0.002) **	
Public specialist	17.6%	(Baseline)
Family doctor	12.1%	0.606 (0.353–1.039)
Private specialist	2.6%	0.101 ** (0.0214–0.479)
**Sex**	Chi^2^ = 0.5 (*p* = 0.481)	
Female	14.2%	(Baseline)
Male	12.4%	1.011 (0.583–1.755)
**Age group**	chi^2^ = 2.2 (*p* = 0.818)	
18–24	9.2%	0.418 (0.130–1.345)
25–34	14.8%	1.583 (0.590–4.243)
35–44	16.0%	0.897 (0.349–2.306)
45–54	14.5%	0.735 (0.266–2.036)
55–64	13.1%	0.764 (0.293–1.991)
65+	12.0%	(Baseline)
**Marital status**	chi^2^ = 1.7 (*p* = 0.192)	
Not married	15.8%	(Baseline)
Married	12.2%	0.822 (0.488–1.384)
**Employment status**	chi^2^ = 0.5 (*p* = 0.489)	
Without a paid job	12.5%	(Baseline)
With a paid job	14.3%	1.758 (0.908–3.404)
**Education**	chi^2^ = 1.6 (*p* = 0.453)	
Primary	15.8%	(Baseline)
Secondary	12.8%	0.770 (0.401–1.479)
Tertiary	11.8%	0.927 (0.451–1.906)
**Income quintile**	chi^2^ = 7.5 (*p* = 0.111)	
1 (lowest)	19.0%	(Baseline)
2	11.6%	0.497 (0.208–1.183)
3	10.8%	0.482 (0.211–1.103)
4	9.2%	0.453 (0.198–1.039)
5 (highest)	17.1%	0.816 (0.332–2.005)
**EQ-5D-5L**	chi^2^ = 1.4 (*p* = 0.244)	
Below median	15.6%	(Baseline)
Above median	12.3%	0.609 (0.343–1.082)
**EQ VAS**	chi^2^ = 3.9 (*p* = 0.049) *	
Below median	16.6%	-
Above median	11.3%	-
**Settlement**	chi^2^ = 3.7 (*p* = 0.155)	
Village	9.0%	(Baseline)
Capital	15.4%	0.515 (0.197–1.345)
Other cities	12.6%	1.542 (0.779–3.052)
**Region**	chi^2^ = 3.3 (*p* = 0.189)	
Central Hungary	12.4%	(Baseline)
Great Plain and North	16.5%	0.720 (0.334–1.554)
Transdanubia	10.8%	0.533 (0.227–1.251)
**Observations**	664	559
**Wald chi^2^ (*p*)**	-	37.68 (*p* = 0.0141)
**Pseudo R^2^**	-	0.0858

OR: odds ratio. Robust 95% confidence intervals (CI) in parentheses. * *p* < 0.05, ** *p* < 0.01, *** *p* < 0.001.

**Table 6:** Waiting time to get an appointment perceived as a problem: share of participants by covariates and multivariate logistic regression results.

VARIABLES	Share of Participants Who Reported Waiting Time to Get an Appointment a Problem	Multivariate Logistic Regression: Was the Time you Waited for the Appointment a Problem for You? OR (95% CI)
Total	25.2%	-
**Waiting time for an appointment**	chi^2^ = 51.5 (*p* < 0.001) ***	
Next day	10.6%	(Baseline)
Within a few days (2–5 days)	8.0%	0.653 (0.133–3.203)
Less than a week (6–7 days)	13.0%	1.648 (0.270–10.08)
Over 1 week (8–14 days)	23.8%	2.050 (0.398–10.56)
Over 2 weeks (15–30 days)	31.8%	8.813 ** (1.892–41.04)
Over 1 month (31–60 days)	42.3%	13.46 ** (2.747–65.90)
Over 2 months (61–90 days)	41.0%	17.69 *** (3.315–94.46)
Over 3 months or more (91 days and more)	55.2%	27.09 *** (4.591–159.8)
**Provider**	chi^2^ = 3.3 (*p* = 0.196)	
Public specialist	27.5%	(Baseline)
Family doctor	25.5%	1.986 (0.882–4.470)
Private specialist	16.9%	0.751 (0.305–1.850)
**Sex**	chi^2^ = 7.4 (*p* = 0.007) ***	
Female	30.3%	(Baseline)
Male	18.5%	0.321 ** (0.156–0.662)
**Age group**	chi^2^ = 5.2 (*p* = 0.395)	
18–24	18.2%	0.757 (0.181–3.163)
25–34	31.5%	2.905 (0.999–8.449)
35–44	27.1%	1.365 (0.425–4.377)
45–54	27.3%	0.645 (0.198–2.097)
55–64	29.0%	1.551 (0.585–4.112)
65+	19.7%	(Baseline)
**Marital status**	chi^2^ = 0.8 (*p* = 0.363)	
Not married	22.4%	(Baseline)
Married	26.5%	1.152 (0.610–2.177)
**Employment status**	chi^2^ = 0.3 (*p* = 0.581)	
Without a paid job	24.1%	(Baseline)
With a paid job	26.5%	2.237 * (1.018–4.917)
**Education**	chi^2^ = 5.1 (*p* = 0.077)	
Primary	32.3%	(Baseline)
Secondary	22.1%	0.778 (0.355–1.702)
Tertiary	21.7%	0.895 (0.373–2.146)
**Income quintile**	chi^2^ = 6.0 (*p* = 0.200)	
1 (lowest)	30.8%	(Baseline)
2	31.5%	1.573 (0.593–4.174)
3	24.6%	0.754 (0.299–1.899)
4	26.3%	2.160 (0.838–5.569)
5 (highest)	15.8%	0.743 (0.273–2.023)
**EQ-5D-5L index**	chi^2^ = 6.5 (*p* = 0.011) *	
Below mean	32.4%	(Baseline)
Above mean	21.1%	0.400 ** (0.210–0.763)
**EQ VAS**	chi^2^ = 7.8 (*p* = 0.005) **	
Below mean	32.0%	-
Above mean	19.9%	-
**Settlement**	chi^2^ = 2.5 (*p* = 0.289)	
Village	22.0%	(Baseline)
Capital	24.2%	1.009 (0.327–3.111)
Other cities	31.7%	0.710 (0.352–1.432)
**Region**	chi^2^ = 1.0 (*p* = 0.598)	
Central Hungary	22.3%	(Baseline)
Great Plain and North	26.7%	1.468 (0.545–3.950)
Transdanubia	27.0%	1.280 (0.465–3.527)
**Observations**	409	353
**Wald chi^2^ (*p*)**	-	65.11 (*p* = 0.0001)
**Pseudo R^2^**	-	0.2413

OR: odds ratio. Robust 95% confidence intervals (CI) in parentheses. * *p* < 0.05, ** *p* < 0.01, *** *p* < 0.001.

**Table 7:** Waiting time at the doctor’s office perceived as a problem: share of participants by covariates and multivariate logistic regression results.

VARIABLES	Share of Participants Who Reported Waiting Time at the Doctor’s Office a Problem	Multivariate Logistic Regression: Was the Time You Waited to Be Seen at a Doctor’s Office a Problem for You? OR (95% CI)
Total	35.5%	-
**Waiting time at the doctor’s office**	chi^2^ = 83.3 (*p* < 0.001 ***)	
Up to half an hour (15–30 min)	11.8%	(Baseline)
Up to an hour (30–60 min)	32.1%	3.753 *** (1.856–7.587)
Between 1 and 2 h	49.2%	8.711 *** (4.314–17.59)
Between 2 and 4 h	57.3%	8.637 *** (3.836–19.45)
Between 4 and 8 h	92.9%	127.4 *** (17.34–936.8)
**Provider**	chi^2^ = 0.2 (*p* = 0.890)	
Public outpatient specialist	34.5%	(Baseline)
General practitioner	36.6%	1.092 (0.656–1.818)
Private outpatient specialist	35.5%	1.166 (0.431–3.156)
**Sex**	chi^2^ = 2.2 (*p* = 0.142)	
Female	38.5%	(Baseline)
Male	32.2%	1.143 (0.682–1.915)
**Age group**	chi^2^ = 20.5 (*p* = 0.001) **	
18–24	42.6%	2.401 (0.851–6.776)
25–34	50.6%	5.329 *** (2.090–13.59)
35–44	40.4%	1.772 (0.718–4.369)
45–54	37.1%	1.561 (0.542–4.497)
55–64	30.9%	1.107 (0.493–2.487)
65+	22.8%	(Baseline)
**Marital status**	chi^2^ = 2.7 (*p* = 0.098)	
Not married	30.6%	(Baseline)
Married	38.1%	1.664 (0.969–2.858)
**Employment status**	chi^2^ = 3.5 (*p* = 0.061)	
Without a paid job	31.7%	(Baseline)
With a paid job	39.7%	1.205 (0.639–2.272)
**Education**	chi^2^ = 0.9 (*p* = 0.627)	
Primary	38.3%	(Baseline)
Secondary	34.8%	0.930 (0.513–1.686)
Tertiary	33.3%	0.733 (0.368–1.461)
**Income quintile**	chi^2^ = 11.8 (*p* = 0.019) *	
1 (lowest)	47.6%	(Baseline)
2	28.6%	0.582 (0.278–1.218)
3	26.7%	0.539 (0.259–1.119)
4	31.2%	0.730 (0.345–1.548)
5 (highest)	37.5%	0.969 (0.426–2.203)
**EQ-5D-5L index**	chi^2^ = 2.1 (*p* = 0.144)	
Below mean	39.6%	(Baseline)
Above mean	33.1%	0.519 * (0.307–0.879)
**EQ VAS**	chi^2^ = 4.3 (0.038) *	
Below mean	40.7%	-
Above mean	31.7%	-
**Settlement**	chi^2^ = 1.7 (*p* = 0.432)	
Village	30.3%	(Baseline)
Capital	37.0%	0.658 (0.260–1.664)
Other cities	36.9%	1.064 (0.600–1.885)
**Region**	chi^2^ = 2.2 (*p* = 0.326)	
Central Hungary	33.3%	(Baseline)
Great Plain and North	39.8%	0.874 (0.394–1.938)
Transdanubia	32.9%	0.770 (0.353–1.680)
**Observations**	501	424
**Wald chi^2^ (*p*)**	-	88.62 (*p* < 0.001)
**Pseudo R^2^**	-	0.2131

OR: odds ratio. Robust 95% confidence intervals (CI) in parentheses. * *p* < 0.05, ** *p* < 0.01, *** *p* < 0.001.

## Data Availability

The data presented in this study are available on request from the corresponding author.

## Supplementary Materials

The following are available online at https://www.mdpi.com/1660-4601/18/5/2213/s1, Table S1: Univariate logistic regression of individuals’ socioeconomic characteristics and waiting times; Table S2: Univariate logistic regression of individuals’ socioeconomic characteristics and perceiving waiting time as a problem.
