# Supplementary material for: Self-Reported Waiting Times for Outpatient Health Care Services in Hungary: Results of a Cross-Sectional Survey on a National Representative Sample
Source: Int J Environ Res Public Health. 2021 Feb 24;18(5):2213. doi: 10.3390/ijerph18052213 (PMC7956329; doi:10.3390/ijerph18052213)
Supplement: Supplementary file 1 [file ijerph-18-02213-s001.pdf]

Online supplement

Table S1. Univariate logistic regression of individuals' socioeconomic characteristics and waiting times.

	**Waited more than 30 days for an appointment with a public specialist**	**Waited more than 2 hours in the doctor's office**
VARIABLES	OR (95% CI)	OR (95% CI)
**Provider**		
Public specialist	–	(Baseline)
Family doctor	–	0.644 (0.406–1.022)
Private specialist	–	0.127 ** (0.0301–0.533)
**Sex**		
Female	(Baseline)	(Baseline)
Male	0.892 (0.544–1.463)	0.850 (0.541–1.337)
**Age group**		
18-24	0.532 (0.192–1.472)	0.746 (0.287–1.941)
25-34	0.414 (0.163–1.048)	1.272 (0.639–2.532)
35-44	0.327** (0.144–0.747)	1.393 (0.713–2.723)
45-54	0.903 (0.415–1.963)	1.239 (0.578–2.660)
55-64	0.824 (0.409–1.660)	1.104 (0.535–2.277)
65+	(Baseline)	(Baseline)
**Marital status**		
Not married	(Baseline)	(Baseline)
Married	0.943 (0.568–1.566)	0.738 (0.467–1.166)
**Employment status**		
Without a paid job	(Baseline)	(Baseline)
With a paid job	0.480 ** (0.283–0.814)	1.171 (0.748–1.832)
**Education**		
Primary	(Baseline)	(Baseline)
Secondary	0.669 (0.368–1.219)	0.780 (0.457–1.332)
Tertiary	0.827 (0.454–1.507)	0.715 (0.411–1.243)
**Income quintile**		
1 (lowest)	(Baseline)	(Baseline)
2	0.494 (0.206–1.183)	0.559 (0.252–1.240)
3	1.185 (0.547–2.570)	0.515 (0.244–1.087)
4	0.293 ** (0.115–0.745)	0.429 * (0.204–0.900)
5 (highest)	0.957 (0.445–2.054)	0.879 (0.448–1.728)
**EQ-5D-5L index**		
Below median	(Baseline)	(Baseline)
Above median	0.886 (0.538–1.459)	0.761 (0.481–1.206)
**Settlement**		
Village	(Baseline)	(Baseline)
Capital	0.740 (0.348–1.574)	0.689 (0.324–1.466)
Other cities	1.175 (0.642–2.150)	1.263 (0.715–2.229)
**Region**		
Central Hungary	(Baseline)	(Baseline)
Great Plain and North	0.774 (0.435–1.377)	1.393 (0.828–2.341)
Transdanubia	1.050 (0.568–1.941)	0.849 (0.467–1.543)

OR: odds ratio. Robust 95% confidence intervals (CI) in parentheses, * $p < 0.05$, ** $p < 0.01$, *** p <

0.001, Constants are not presented.

Table S2. Univariate logistic regression of individuals' socioeconomic characteristics and perceiving waiting time as a problem.

	**Was the time you waited for the appointment a problem for you?**	**Was the time you waited to be seen at a doctor's office a problem for you?**
VARIABLES	OR (95% CI)	OR (95% CI)
**Waiting time for an appointment**		
Next day	(Baseline)	
Within a few days (2-5 days)	0.726 (0.217–2.430)	
Less than a week (6-7 days)	1.260 (0.356–4.464)	
Over 1 week (8-14 days)	2.625 (0.815–8.452)	
Over 2 weeks (15-30 days)	3.920 * (1.354–11.35)	
Over 1 month (31-60 days)	6.160 *** (2.093–18.13)	
Over 2 months (61-90 days)	5.843 ** (1.893–18.03)	
Over 3 months or more (91 days and more)	10.34 *** (3.168–33.74)	
**Waiting time at the doctor's office**		
Up to half an hour (15–30min)		(Baseline)
Up to an hour (30–60 min)		3.526 *** (1.922–6.471)
Between 1 and 2 hours		7.200 *** (3.916–13.24)
Between 2 and 4 hours		10.00 *** (5.106–19.60)
Between 4 and 8 hours		96.78 *** (11.91–786.1)
**Provider**		
Public specialist	(Baseline)	(Baseline)
Family doctor	0.906 (0.526–1.559)	1.097 (0.752–1.602)
Private specialist	0.537 (0.272–1.063)	1.046 (0.477–2.291)
**Sex**		
Female	(Baseline)	(Baseline)
Male	0.523 ** (0.327–0.839)	0.759 (0.525–1.098)
**Age group**		
18-24	0.907 (0.338–2.435)	2.509 * (1.241–5.073)
25-34	1.877 (0.970–3.633)	3.472 *** (1.920–6.278)
35-44	1.520 (0.766–3.017)	2.298 ** (1.293–4.085)
45-54	1.530 (0.690–3.390)	1.998 * (1.039–3.839)
55-64	1.669 (0.827–3.369)	1.512 (0.814–2.809)
65+	(Baseline)	(Baseline)
**Marital status**		
Not married	(Baseline)	(Baseline)
Married	1.253 (0.770–2.039)	1.395 (0.940–2.070)
**Employment status**		
Without a paid job	(Baseline)	(Baseline)
With a paid job	1.134 (0.725–1.776)	1.419 (0.983–2.050)
**Education**		
Primary	(Baseline)	(Baseline)
Secondary	0.593 (0.343–1.026)	0.859 (0.555–1.331)
Tertiary	0.580* (0.337–0.997)	0.805 (0.508–1.273)

**Income quintile**		
1 (lowest)	(Baseline)	(Baseline)
2	1.034 (0.488–2.189)	0.441 * (0.231–0.842)
3	0.736 (0.355–1.526)	0.402 ** (0.217–0.744)
4	0.804 (0.398–1.622)	0.499 * (0.278–0.897)
5 (highest)	0.422 * (0.193–0.923)	0.661 (0.358–1.223)
**EQ–5D-5L index**		
Below median	(Baseline)	(Baseline)
Above median	0.556 * (0.353–0.877)	0.756 (0.519–1.101)
**Settlement**		
Village	(Baseline)	(Baseline)
Capital	0.607 (0.307–1.199)	0.743 (0.419–1.317)
Other cities	0.686 (0.394–1.192)	1.006 (0.631–1.603)
**Region**		
Central Hungary	(Baseline)	(Baseline)
Great Plain and North	1.267 (0.745–2.154)	1.321 (0.853–2.047)
Central Hungary	1.291 (0.729–2.285)	0.980 (0.615–1.563)

OR: odds ratio. Robust 95% confidence intervals (CI) in parentheses, * $p < 0.05$, ** $p < 0.01$, *** $p < 0.001$, Constants are not presented.
